# The effect of AKT inhibition in α-synuclein-dependent neurodegeneration

**DOI:** 10.3389/fnmol.2025.1524044

**Published:** 2025-02-05

**Authors:** Bedri Ranxhi, Zoya R. Bangash, Zachary M. Chbihi, Sokol V. Todi, Peter A. LeWitt, Wei-Ling Tsou

**Affiliations:** ^1^Department of Pharmacology, Wayne State University School of Medicine, Detroit, MI, United States; ^2^Department of Neurology, Wayne State University School of Medicine, Detroit, MI, United States; ^3^Department of Neurology, Henry Ford Health Systems, Detroit, MI, United States

**Keywords:** α-synuclein, synucleinopathy, AKT, NF-κB, neurodegenerative disease, Parkinson’s disease, proteinopathy, protein misfolding disorders

## Abstract

Parkinson’s disease (PD) is a progressive neurodegenerative disorder affecting millions of individuals worldwide. A hallmark of PD pathology is the accumulation of α-synuclein (α-Syn), a small protein known to support neuronal development and function. However, in PD, α-Syn cumulatively misfolds into toxic aggregates that disrupt cellular processes and contribute to neuronal damage and neurodegeneration. Previous studies implicated the AKT signaling pathway in α-Syn toxicity in cellular models of PD, suggesting AKT as a potential therapeutic target. Here, we investigated the effect of AKT inhibition in a *Drosophila* model of synucleinopathy. We observed that administration of the AKT inhibitor, A-443654 led to mild improvements in both survival and motor function in flies expressing human α-Syn. Genetic studies revealed that reduction of AKT levels decreased α-Syn protein levels, concomitant with improved physiological outcomes. The protective effects of AKT reduction appear to operate through the fly ortholog of NF-κB, Relish, suggesting a link between AKT and NF-κB in regulating α-Syn levels. These findings highlight the AKT cascade as a potential therapeutic target for synucleinopathies and provide insights into mechanisms that could be utilized to reduce α-Syn toxicity in PD and related disorders, such as multiple system atrophy.

## Introduction

Parkinson’s Disease (PD) is a progressive neurodegenerative disorder marked by the degeneration of dopaminergic neurons in the substantia nigra (Louis et al., 2022; Dauer and Przedborski, 2003). The abnormal accumulation and aggregation of α-synuclein (α-Syn), which is the primary component of Lewy body fibrils (Spillantini et al., 1997), is a key pathological feature in PD and related synucleinopathies (Goedert et al., 2017). Mutation of *SNCA*, the gene encoding α-Syn, Causes autosomal dominant Parkinsonism (Bridi and Hirth, 2018). Other genetic evidence includes *SNCA* gene multiplications, which lead to another Mendelian-inherited form of Parkinsonism (Bridi and Hirth, 2018). The increased intraneuronal production of wild-type α-Syn as a causative factor for Parkinsonism supports the idea of a toxic gain-of-function resulting from excess α-Syn protein (Garcia-Reitbock et al., 2010; Cuervo et al., 2004; Benskey et al., 2016). Furthermore, α-Syn, a protein involved in synaptic vesicle trafficking (Calabresi et al., 2023; Gomez-Benito et al., 2020), loses its normal physiological function and becomes toxic as it misfolds and aggregates, leading to motor dysfunction and other PD-related symptoms. Key mechanisms triggering α-Syn aggregation and its resultant toxicity include oxidative stress, mitochondrial dysfunction (Rocha et al., 2018; Hu et al., 2019), and impaired autophagy (Tanik et al., 2013), all of which contribute to neuronal degeneration in PD. These findings suggest that reducing the overall expression of α-Syn is a promising therapeutic approach for PD and related disorders.

The AKT pathway plays an important role in regulating both transcription and protein degradation, processes that are essential for maintaining cellular homeostasis (Brunet et al., 2001; Glaviano et al., 2023). The AKT pathway is commonly activated in various human pathologies, including cancers and neurodegenerative disorders such as PD (Nitulescu et al., 2018; Hers et al., 2011). In PD, the PI3K/AKT signaling pathway has drawn interest as a potential therapeutic target due to a major role in promoting cell survival and modulating mechanisms implicated in neurodegeneration (Dong-Chen et al., 2023). As a central component of the PI3K/Akt/mTOR signaling pathway, AKT regulates cell survival, growth, and apoptosis (Brunet et al., 2001; Peng et al., 2022). However, the specific effects exerted by the AKT pathway on α-Syn and its accumulation remain unclear, requiring further investigation to understand its role in α-Syn-related toxicity and aggregation in PD.

A-443654 (Han et al., 2007; Luo et al., 2005) is a potent and selective AKT inhibitor derived from indazole–pyridine compounds; it targets all three AKT isoforms (AKT1, AKT2, and AKT3) by binding to the ATP-binding site, acting as an ATP-competitive and reversible inhibitor. A-443654 has been studied for its potential therapeutic applications in treating various diseases, including glioblastoma multiforme (Gallia et al., 2009) and PD (Gandelman et al., 2021). In cellular models of PD, A-443654 significantly reduces α-Syn protein levels, effectively alleviating α-Syn toxicity and restoring cellular function in ATXN2-Q58 cells. This recovery was associated with the normalization of critical autophagy and stress response markers, including mTOR, LC3-II, p62, STAU1, BiP, and CHOP (Gandelman et al., 2021). Furthermore, A-443654 successfully restored normal α-Syn levels in both fibroblasts and iPSC-derived dopaminergic neurons from a patient with a triplication of the *SNCA* gene (Gandelman et al., 2021). Building on these findings from cellular models, our study sought to determine whether similar effects of A-443654 can be observed in an intact organism. We utilized a *Drosophila melanogaster* model of PD that overexpresses the human SNCA gene pan-neuronally, driven by the elav-Gal4 driver. This approach was chosen not only due to the broad impact of PD on the nervous system more generally, but also because of the historical use of this pattern of expression to model synucleinopathies in the fruit fly (Feany and Bender, 2000; Takahashi et al., 2003; Mohite et al., 2018), which will enable our findings to be placed in the context of other investigations using the same models. We found that A-443654 administration could alleviate α-Syn-induced toxicity by extending fly longevity and improving mobility. Genetic reduction of AKT expression led to a decrease in α-Syn protein level, whereas increased AKT expression led to its elevation. Of interest was that manipulation of NF-κB, a downstream target of AKT, via knockdown or overexpression, also impacted α-Syn levels, indicating that NF-κB itself may play a critical role in the regulation of α-Syn. These findings provide valuable insights into the possibility of targeting the AKT pathway as a therapeutic strategy for PD and for other α-synucleinopathies, including the glial pathology observed in multiple system atrophy (Spillantini et al., 1997; Sokratian et al., 2024).

## Materials and methods

### Fly stocks and maintenance

elav-Gal4 (#458), UAS-α-Syn^WT^ (#51374), UAS-Ataxin-2 (#68395), UAS-AKT^RNAi^ (#31701), UAS-Rel^RNAi^ (#28943), UAS-AKT (#8191), and UAS-Rel (#9459) were obtained from the Bloomington *Drosophila* Stock Center. Gifted stocks used in this study were y,w; +; attP2 (Jamie Roebuck, Duke University) and w^1118^ (Russ Finley, Wayne State University). UAS-ATXN3(Q80) was previously described in Blount et al. (2023), Prifti et al. (2022), and Johnson et al. (2020). Flies were housed in a 25°C incubator on a 12-h light and 12-h dark cycle at ~40% relative humidity. Control flies for all Gal4-UAS experiments consisted of the elav-Gal4 line crossed to y,w; +; attP2 or w^1118^ flies, depending on experiment. Adult offspring were synchronized by collecting within 12 h of eclosion over a 48-h period. Groups of 20 age- and sex-matched flies were immediately transferred into narrow polypropylene vials containing 5 mL of standard 2% agar, 10% sucrose, 10% yeast with appropriate preservatives. Food vials were changed every second to third day.

### Supplementation of A-443654 and rotenone on fly media

A-443654 (Advanced ChemBlocks Inc., CA, United States) was initially dissolved in dimethylsulfoxide (DMSO) to establish a 50 mM stock solution. The stock was then diluted with water to final concentrations of 100 μM and 1,000 μM, as indicated in the respective experiments. These concentrations were selected based on treatment protocols from previous publications (Johnson et al., 2015). To prepare A-443654-treated fly food, 100 μL of the diluted A-443654 solution was added to the top of 5 mL of fly food, which was then allowed to air-dry before transferring flies into the vial. For the preparation of rotenone fly food, a 100 mM stock solution of rotenone (Sigma-Aldrich, St. Louis, MO, United States) was prepared by dissolving rotenone in DMSO. A working solution of 500 μM rotenone was then made by diluting the stock solution with water. To prepare the fly food, 100 μL of the 500 μM rotenone solution was added to the top of 5 mL of fly food, followed by air drying prior to transferring the flies. The rotenone concentration was selected based on reports from previous studies (Coulom and Birman, 2004; Akinade et al., 2022). For fly food containing both A-443654 and rotenone, 100 μL of either the 100 µM or 1,000 μM A-443654 solution was added to the prepared rotenone-treated fly food. To prepare control food, 100 μL of a DMSO/water solution (containing the same volume of DMSO as used for A-443654 with or without rotenone dilutions) was added to 5 mL of fly food. This was done to match the DMSO concentration used in the experimental groups. The food was allowed to air-dry before transferring the flies into the vial.

### Lifespan

At least 200 adults (100 adults in the longevity test in Supplementary Figures) were age-matched and separated by sex within 48 h of eclosion. Every 2–3 days, flies were transferred to a new vial with food with the indicated drug in each experiment; dead flies were counted until none remained. Survival curves were analyzed by log-rank in GraphPad Prism (San Diego, CA, United States).

### RING assay

Negative geotaxis was evaluated using a modified Rapid Iterative Negative Geotaxis (RING) assay in groups of at least 100 flies, following the procedure outlined in a prior publication (Gargano et al., 2005). Briefly, 5 vials containing 20 flies each were briskly tapped to the bottom, and their climbing distance was captured in a photograph (Sony, Tokyo, Japan) 3 s after initiating the flies’ natural geotaxis response. For each group, an average of 5 consecutive trials was recorded. Flies were tested longitudinally once per week, and between tests they were returned to standard food vials and housed as described above. Negative geotaxis performance was analyzed by scoring the position of each fly within designated zones of the vial. Based on an established protocol, the fly distribution within each zone was then converted to percentages using RStudio (Boston, MA, United States) (Gargano et al., 2005; Richardson et al., 2024). All negative geotaxis experiments were performed in duplicate, with one complete trial shown in each graph.

### Western blots

Fourteen fly heads (7 females and 7 males) per biological replicate were homogenized in boiling lysis buffer containing 50 mM Tris (pH 6.8), 2% SDS, 10% glycerol, and 100 mM dithiothreitol. The homogenates were sonicated, boiled for 10 min, and centrifuged at 13,300 g for 10 min at room temperature. Western blots were performed using at least 3 biological replicates per group. Fly lysates were separated by electrophoresis using a 4–20% Mini-PROTEAN^®^ TGX™ Precast Gel and transferred onto a 0.2 μm PVDF membrane (Bio-Rad, Hercules, CA, United States). Membranes were blocked for 30 mins at room temperature in 10 mM Tris–HCl (pH 8.0), 150 mM NaCl, and 0.1% Tween-20, supplemented with 5% milk solids. Following blocking, membranes were incubated overnight with primary antibodies and 1 h in secondary antibodies the following day. Western blots were developed using either EcoBright Pico HRP or Femto HRP 100 (Innovative Solutions, MI, United States) and imaged using a ChemiDoc system (Bio-Rad, Hercules, CA, United States).

### Antibodies, direct blue staining, and protein quantification

The primary antibodies used were mouse anti-α-Syn (4B12) (1:1,000, Sigma-Aldrich, St. Louis, MO, United States), rabbit anti-AKTpan (C67E7) (1:1,000, Cell Signaling, Danvers, MA, United States), and mouse anti-Relish-C (21F3) (1:500, Developmental Studies Hybridoma Bank, IA, United States). The secondary antibodies included peroxidase-conjugated anti-mouse and peroxidase-conjugated anti-rabbit (1:5,000, Jackson ImmunoResearch, West Grove, PA, United States). For total protein normalization using Direct Blue 71 staining, PVDF membranes were submerged for 10 min in 0.01% Direct Blue 71 (Sigma-Aldrich, St. Louis, MO, United States) dissolved in a solution of 40% ethanol and 10% acetic acid, then rinsed in 40% ethanol/10% acetic acid, air-dried, and imaged. α-Syn protein levels were quantified by measuring the band at approximately 17 kDa. In the gene overexpression or RNAi groups, α-Syn levels were normalized to the control group, with total protein content for each genotype ascertained using Direct Blue 71 staining. Band quantification was performed using ImageLab (Bio-Rad, Hercules, CA, United States).

### Statistics

Prism 9 (GraphPad) was used for graphics and statistical analyses. Statistical analyses used are noted in the figure legends.

## Results

### The effect of A-443654 α-Syn-dependent toxicity in *Drosophila* neurons

**Figure 1:**
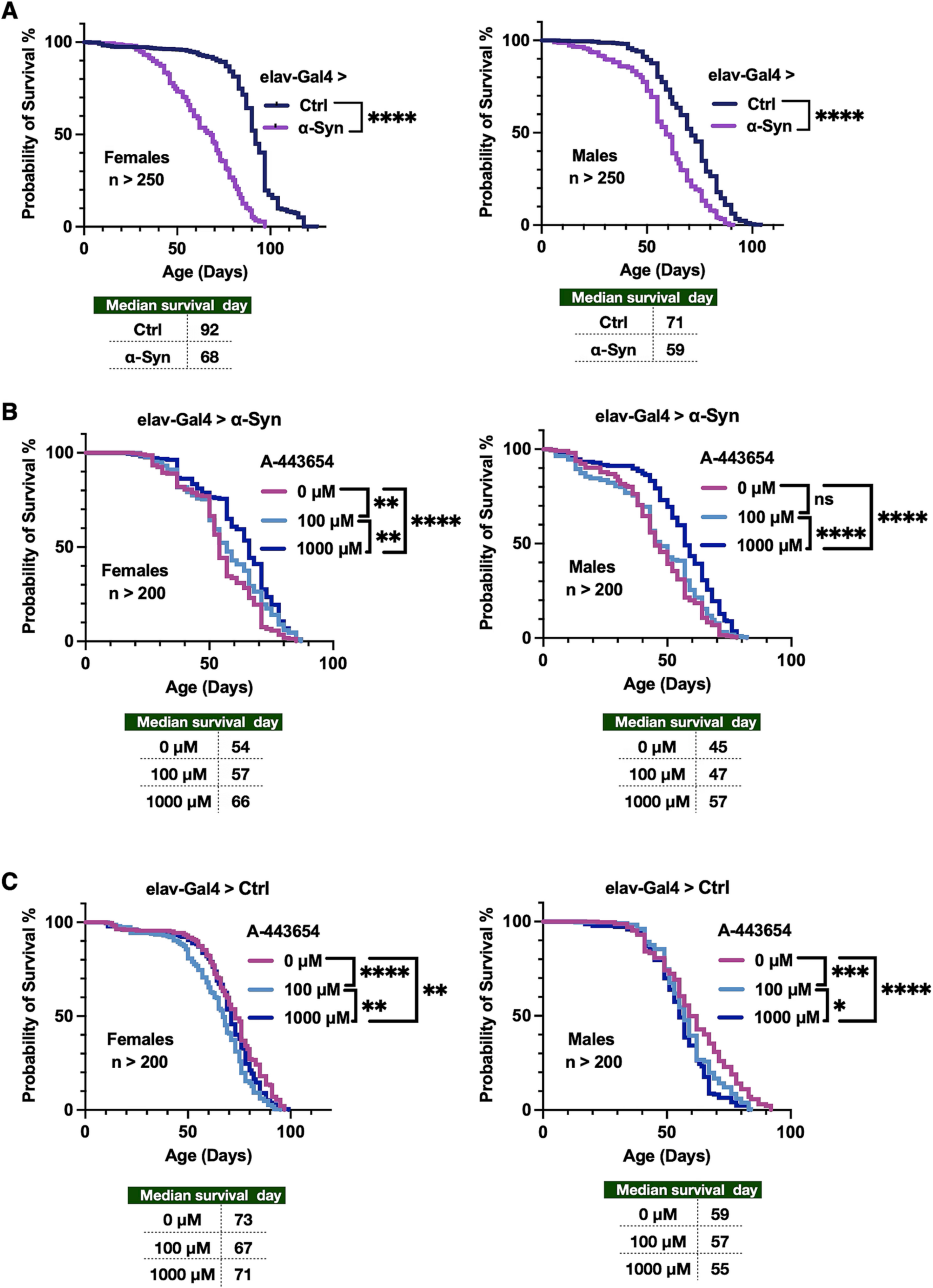
Effect of A-443654 on the longevity of flies pan-neuronal expressing α-Syn. **(A)** Longevity graphs of adult female and male flies expressing α-Syn in all fly neurons, throughout development and in adults. Driver was elav-Gal4. Statistics: log-rank tests. **(B)** Longevity of adult flies as in **(A)** expressing α-Syn, fed with the compound A-443654 or its vehicle, starting as day 1 adults and for the rest of their lives. Statistics: log-rank tests. **(C)** Longevity graphs of flies not expressing any α-Syn, fed A-443654 or its vehicle, starting as day 1 adults and for the rest of their lives. Statistics: log-rank tests. No significant difference is denoted as (ns), *p* < 0.05 is denoted as (*), *p* < 0.01 is denoted as (**), *p* < 0.001 is denoted as (***) and *p* < 0.0001 is denoted as (****). *N* > 200 per group.

To examine any effect from A-443654 on toxicity from the presence of α-Syn in the nervous system of the fruit fly, we began by expressing human α-Syn in *Drosophila* neurons using the Gal4-UAS system (Brand and Perrimon, 1993). Gal4-UAS allows for specific expression of various transgenes in the tissues and timeframes of interest. In our case, we expressed human, wild-type UAS-α-Syn in all fly neurons throughout development and in adulthood by using the driver elav-Gal4. As shown in Figure 1A, expression of human α-Syn in all fly neurons led to reduced overall longevity in both male and female flies.

Next, we tested whether adding A-443654 to the fly media could improve the reduced longevity observed in adult flies that pan-neuronally express α-Syn. For these assays, flies were reared on media without A-443654 during the embryonic, larval, and pupal stages. On the first day of adulthood, upon eclosion from the pupal case, male and female flies were separated and transferred onto media supplemented with A-443654 at concentrations of 0 µM, 100 µM, or 1,000 μM. Flies were transferred to new vials with fresh media containing A-443654 every other day. As shown in Figure 1B, addition of 100 μM A-443654 extended the longevity of female flies expressing α-Syn, but had no effect on male flies. However, addition of 1,000 μM A-443654 led to increased overall longevity in both sexes. As shown in Figure 1C, control flies with the Gal4 driver in the absence of α-Syn had overall reduced longevity when they were fed either of the A-443654 concentrations; this decrease may be due to the inherent toxicity of A-443654 in flies (Luo et al., 2005). Addition DMSO vehicle reduced control fly longevity, suggesting potential vehicle toxicity.

**Figure 2:**
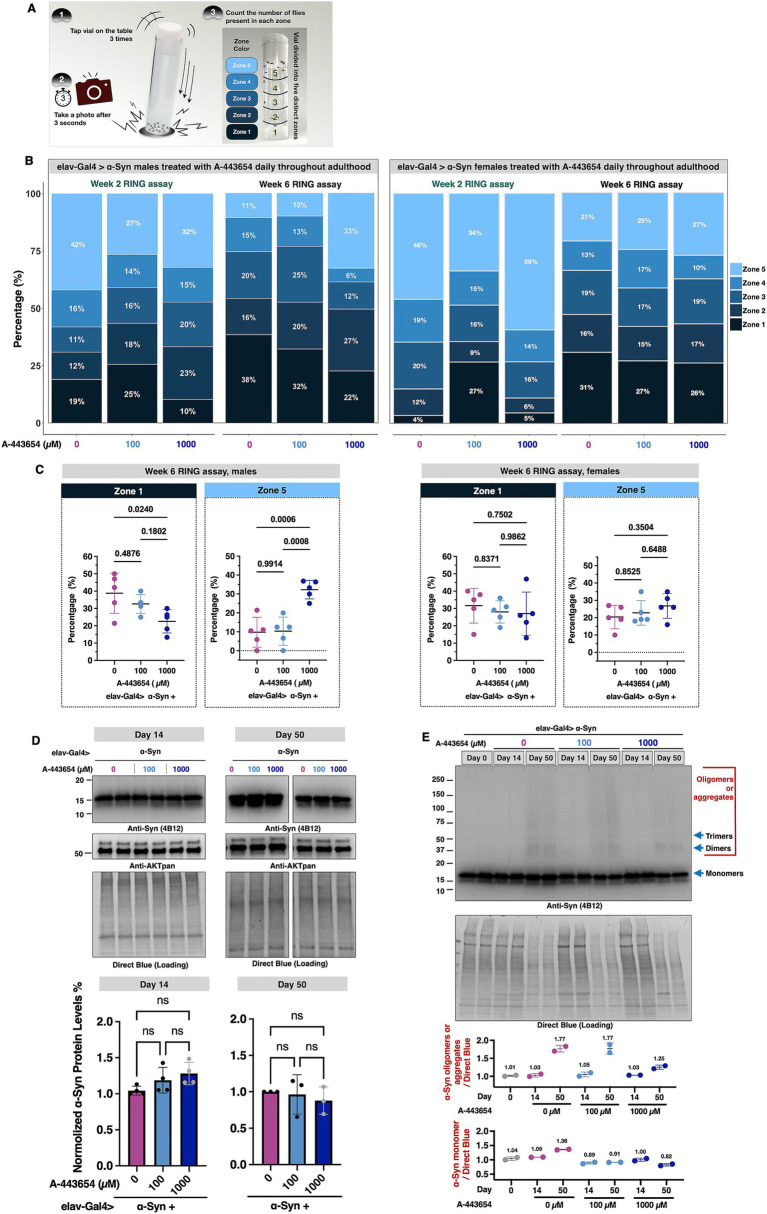
Effect of A-443654 on the motility and α-Syn protein levels in flies with pan-neuronal α-Syn expression. **(A)** Diagram illustrating methodology of the Rapid Iterative Negative Geotaxis (RING) assay. For details, please see the “Materials and methods.” **(B)** Graphical representation of the proportion of adult male (left) and female (right) flies present in each of the zones of the vial in RING assay. N ≥ 100 flies per group. **(C)** Zone 1- and zone 5-specific comparisons conducted for week 6 flies across A-443654-treated groups. The percentage of flies in each zone was calculated for individual vials, with each group comprising 5 vials containing 20 flies per vial. Statistical analysis was performed using one-way ANOVA followed by Tukey’s *post-hoc* test in GraphPad Prism. **(D)** Top panel: Western blots and related quantifications of the levels of α-Syn and total AKT in the absence or presence of A-443654 treatment in adult flies at days 14 and 50. Fourteen fly heads (7 females and 7 males) per biological replicate were used. At least 3 biological replicates were conducted per group. Bottom panel: graph of α-Syn protein levels quantified from the top and other independent repeats. Statistical analysis: Kruskal-Wallis test followed by Dunnett’s *post-hoc* test was performed for multiple group comparisons. ns: not statistically significant. **(E)** Western blots of α-Syn in flies treated with the indicated concentrations of A-443654 at days 0, 14, and 50. Two biological replicates were conducted per group. Blue arrows indicate α-Syn monomer, dimer, and trimer bands based on their respective molecular weights. Regions corresponding to oligomers or aggregates are highlighted in red. The quantifications of oligomer/aggregate and monomer protein levels are presented in the bottom panels. The numbers above the data points represent the mean values for each group.

Next, we assessed the ability of A-443654 to modulate another aspect of fly physiology, their mobility. Figure 2A depicts the RING assay (Gargano et al., 2005) that we performed on flies pan-neuronally expressing α-Syn. We evaluated flies at weeks 2 and 6, chosen to examine the mobility of young flies as well as at a time point when we observed effects from 1,000 μM administration in longevity (Figure 2B). In week 2, we observed that administration of 100 μM A-443654 led to a higher preponderance of male and female flies in the bottom range of the zones, indicating reduced mobility, compared to flies fed with the vehicle (Figure 2B). Administration of 1,000 μM A-443654 led to fewer male and female flies in zone 1 and higher numbers in zone 2 compared to the vehicle control group. However, while male flies showed fewer individuals in zone 5 compared to controls, female flies exhibited a higher presence in zone 5 than their respective controls. In week 6, 100 μM A-443654 appeared to mildly improve mobility, with more males in zones 2 and 3 and more females in zones 4 and 5 compared to their respective controls. Meanwhile, 1,000 μM A-443654 led to a marked reduction in the proportion of flies in zone 1 and an increase proportion in zones 2 and 5 for both males and females (Figure 2B). To further analyze the locomotor response in the lowest- and highest-performing groups, we assessed the distribution of week 6 flies in the bottom (zone 1) and top (zone 5) of the vials, calculating the percentage of flies treated with 0 μM, 100 μM, and 1,000 μM A-443654 in each zone. In males, treatment with 1,000 μM A-443654 significantly reduced the number of flies in zone 1 and increased their presence in zone 5, indicating improved locomotor response (Figure 2C, left). In contrast, females did not show significant changes in zones 1 or 5 across the different A-443654 treatment groups (Figure 2C, right). These findings suggest that A-443654 mildly but significantly enhances locomotor function in male flies expressing α-Syn at week 6, but has no notable effect on their female siblings.

Lastly, we examined whether feeding A-443654 influences the protein levels of α-Syn and AKT. We homogenized flies from the same groups as described in Figure 2B at two points, days 14 and 50. Neither dose of A-443654 led to a detectable change in the total protein levels of α-Syn and AKT (Figure 2D). This result aligns with the mechanism of A-443654, which is designed to inhibit AKT enzymatic activity without changing its protein level (Luo et al., 2005; Shi et al., 2005). When comparing α-Syn protein levels between day 14 and day 50, we observed an increase in higher molecular weight α-Syn species (oligomers and aggregates) at day 50 (Figure 2E). Flies treated with 1,000 μM A-443654 exhibited reduced levels of higher molecular weight α-Syn compared to the 0 μM and 100 μM groups. These findings suggest that 1,000 μM A-443654 enhances the longevity and mobility of flies expressing α-Syn by potentially by reducing the accumulation of aggregated α-Syn, as assessed from Western blotting.

### The effect of A-443654 on other models of degeneration in *Drosophila*

**Figure 3:**
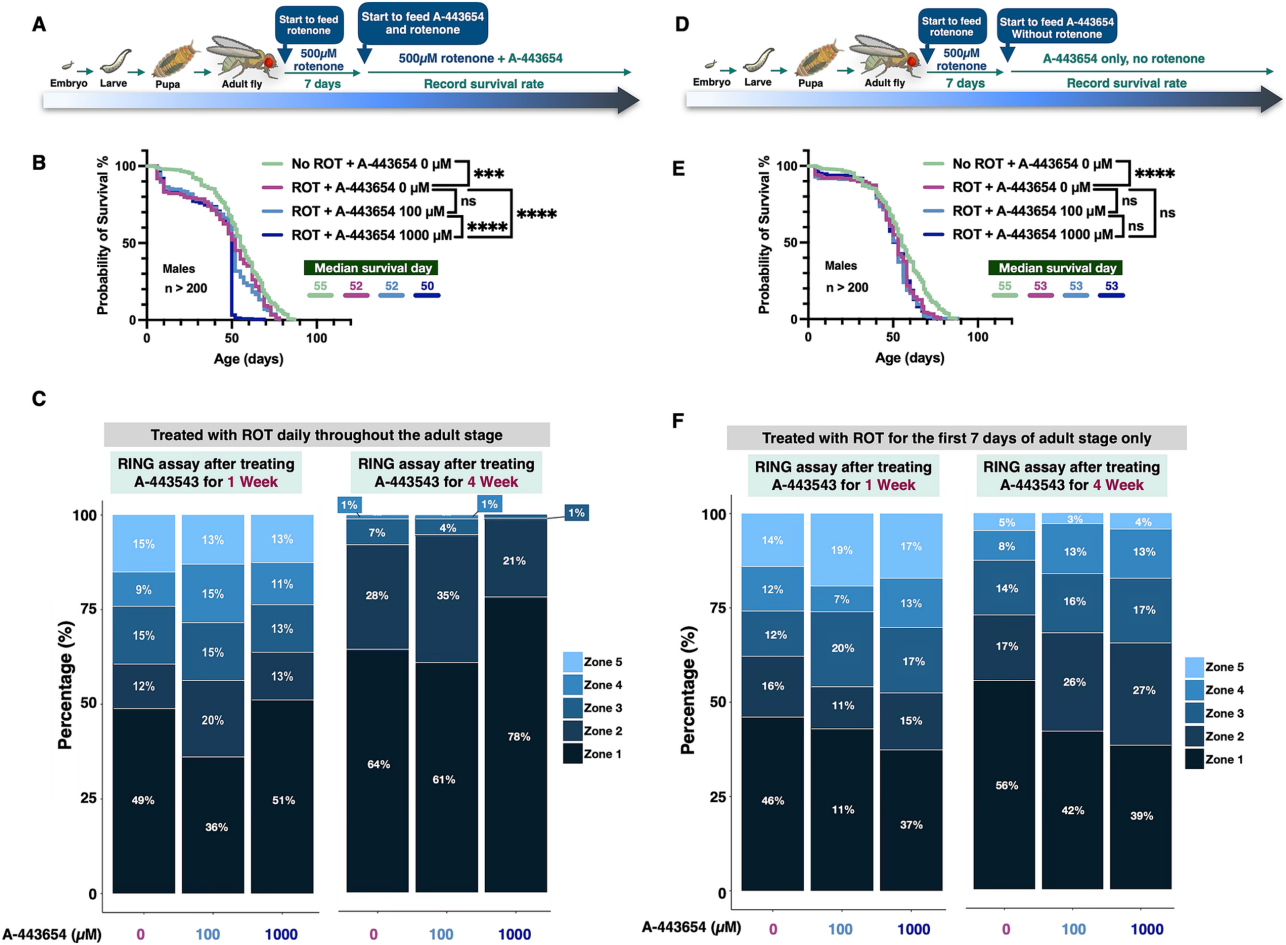
The impact of A-443654 on ROT-treated male flies. **(A,D)** Schematics illustrating the rotenone (ROT)-feeding experiment. In both panels, ROT was administered continuously from adult fly eclosion throughout adulthood **(A)** or halted on day 7 of adult fly life **(D)**, with A-443654 introduced in the food at the indicated concentrations starting on day 7 and continuing throughout adulthood. Longevity outcomes are shown in panels **B** and **E**. Statistical analyses were performed using the log-rank test; no significant difference is denoted as (ns), *p* < 0.05 as (*), *p* < 0.01 as (**), *p* < 0.001 as (***) and *p* < 0.0001 as (****). *N* > 200 per group. **(C,F)** Motility outcomes from flies treated with ROT continuously **(C)** or for 7 days only **(F)**. N ≥ 100 flies per group, collected from 5 individual vials.

**Figure 4:**
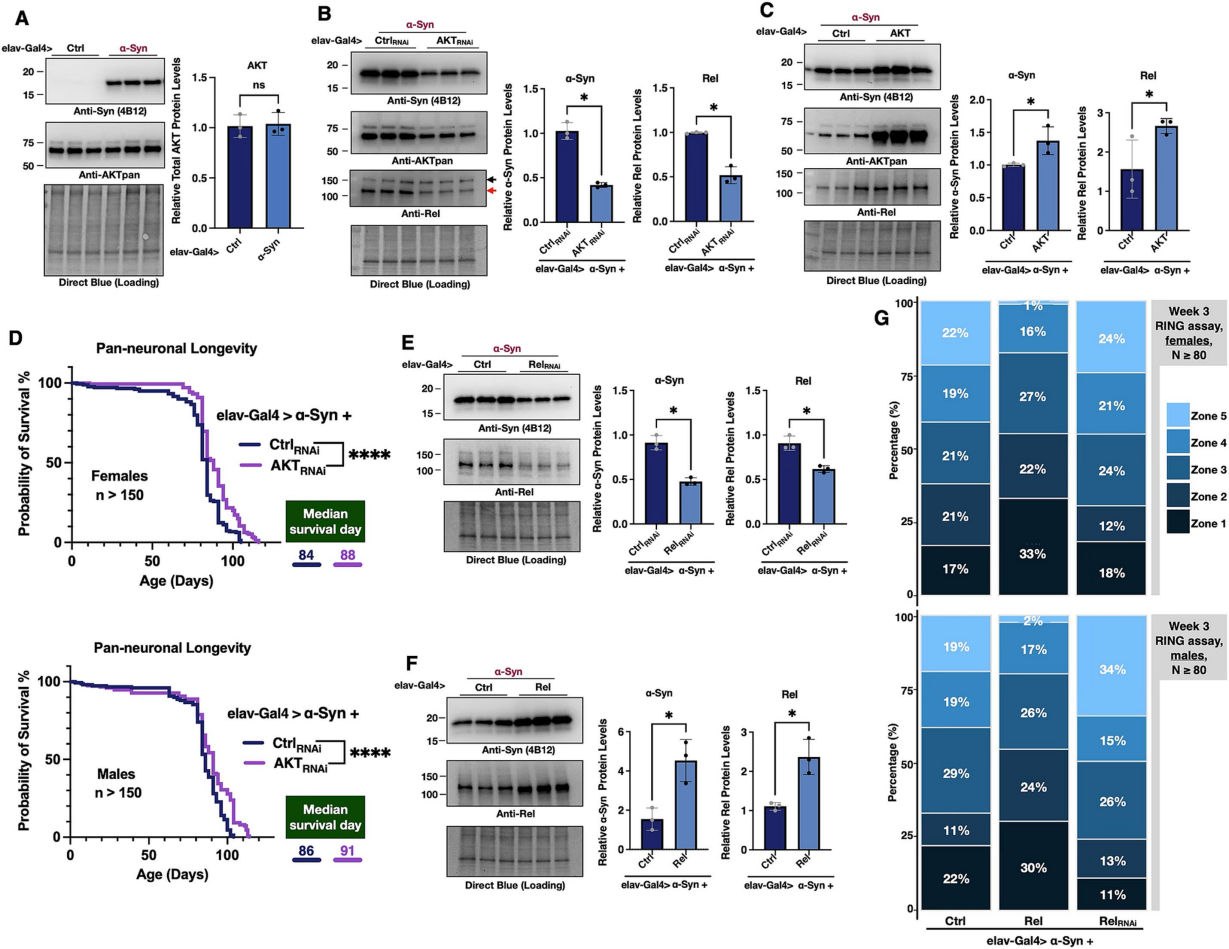
The impact of AKT and Relish on α-Syn protein levels. **(A–C,E,F)** Western blots of the indicated proteins. α-Syn was expressed pan-neuronally in flies using the elav-Gal4 driver either alone **(A)** or in combination with AKT knockdown **(B)**, AKT overexpression **(C)**, Relish (Rel) knockdown **(E)**, or Rel overexpression **(F)**. Symbols to the right of the bands in **(B)** indicate Rel (red arrow) and non-specific bands (black arrow). In the corresponding graphs, protein quantification from images was normalized to their respective Direct Blue loading controls. All lanes were further normalized to lane 1 to determine the relative expression levels. *N* = 3 biological replicates per group. Data are presented as means ± SD. Statistical analyses: Mann–Whitney one-tailed test. ns: no significance, * (*p* < 0.05). **(D)** Longevity of adult female and male flies pan-neuronally expressing α-Syn with Ctrl or AKT knockdown. Statistical analysis: log-rank test, **** (*p* < 0.0001). *N* > 150 per group. **(G)** RING assay results for week-3 female (top) and male (bottom) flies pan-neuronally expressing α-Syn with control, Rel overexpression, or Rel knockdown. *N* ≥ 80 flies per group.

To investigate if the effect from A-443654 is specific to the α-Syn model, or if it might apply more generally to other disease models, we tested the outcomes of 100 µM and 1,000 μM A-443654 on flies exposed to rotenone, which models PD through mitochondrial toxicity (Saravanan et al., 2005; Girish and Muralidhara, 2012). We also tested the effect of this AKT inhibitor on the toxicity of non-PD neurodegenerative proteins that form aggregates due to polyglutamine expansion mutations specifically, ataxin-2 (associated with spinocerebellar ataxia type 2, SCA2; Pulst et al., 1996) and ataxin-3 (associated with spinocerebellar ataxia type 3, SCA3; Paulson et al., 1997). Through its action on mitochondria, rotenone damages dopaminergic neurons and is widely used in rodent models to induce Parkinsonian signs (Rocha et al., 2022; Akinade et al., 2022; Coulom and Birman, 2004). We examined how A-443654 affects flies fed with rotenone. For rotenone exposure, we carried out two different treatment protocols. One of them (Figure 3A) involved feeding flies with rotenone throughout their lifetime; after 7 days as adults, A-443654 was introduced. As shown in Figure 3B, continued rotenone feeding led to reduced adult fly longevity (Supplementary Figure 1A for female fly longevity). Co-administration of 100 μM A-443654 did not yield any detectable benefit, whereas feeding of 1,000 μM A-443654 enhanced toxicity. Notably, the addition of the DMSO vehicle, used to dissolve both A-443654 and rotenone, led to reduced longevity in control flies compared to those not exposed to DMSO (Figure 1A) or exposed to lower levels of DMSO (Figure 1C). Motility assays in both week 1 and week 4 showed a slight improvement in the mobility of flies fed rotenone and then 100 μM A-443654 treatment when the proportion of flies was compared at the lowest zone (Figure 4C). Feeding of 1,000 μM A-443654 did not lead to an appreciable difference from control flies, which were fed rotenone but not A-443654. After 4 weeks of rotenone feeding, over 60% of the flies remained in the bottom zone. Neither 100 µM nor 1,000 μM of A-443654 improved their motility; the higher dose of 1,000 μM worsened motility (Figure 3C).

Given the toxic effects of lifetime rotenone feeding (reduced longevity and motility), we next limited rotenone treatment to just 7 days in adult flies (Girish and Muralidhara, 2012). After being exposed to rotenone, flies were switched to regular food containing either 0 µM, 100 µM, or 1,000 μM A-443654 (Figure 3D). In this experiment, rotenone again reduced fly longevity. Neither A-443655 100 μM or 1,000 μM concentrations had any effect (Figure 3E; Supplementary Figure 1B for female fly longevity). However, in motility assays feeding of either concentration led to a smaller proportion of flies in the bottom-most zone compared to flies not fed A-443654 at two different time points tested (Figure 3F). Collectively, these results suggest that A-443654 preserves mobility in the rotenone-based fly model of PD, but it does not enhance fly longevity – in fact, in the more severe model (Figure 3B), A-443654 exacerbated death.

The potentially protective effect from A-443654 does not appear confined solely to PD models with pan-neuronal expression of α-Syn in *Drosophila*: when we tested its ability to modify reduced longevity from two non-PD-related proteins (ataxin-2 and ataxin-3), we observed a very mild, but statistically significant, positive effect from A-443654 in these models of SCA2 and SCA3 (Supplementary Figure 2). Altogether, we conclude that A-443654 treatment can provide some mild protection in several models of proteinopathy-associated neurodegenerative diseases.

### Targeting AKT through *Drosophila* genetics implicates it and NF-kB in α-Syn toxicity

As noted above, we observed a protective effect from A-443654 administration on the survival rate and motility of flies pan-neuronally expressing α-Syn. Next, we turned our attention to fly genetics to investigate relationships between the targets of A-443654, AKT, and α-Syn. To begin with, we examined if the presence of α-Syn changes overall levels of AKT in the fly. We did not observe a difference in total AKT protein levels when α-Syn was expressed in all fly neurons (Figure 4A). Next, we used genetics to reduce AKT levels through using RNA-interference (RNAi), or to exogenously promote its over-expression in the same tissues. We found that reducing AKT expression significantly lowered α-Syn protein levels in fly neurons (Figure 4B), whereas AKT overexpression led to increased α-Syn protein levels (Figure 4C). Concomitantly with reduced levels of α-Syn in fly neurons when AKT was knocked down, we also observed increased longevity in both female and male flies (Figure 4D). This finding suggests a potential physiologically relevant outcome for PD achievable from a reduction in AKT levels in neurons co-expressing α-Syn.

Because one of the downstream targets of AKT is NF-κB, known in *Drosophila* as Relish (Rel), we also probed for the levels of Rel in this setup. We observed a reduction in endogenous Rel protein levels when AKT was downregulated using RNAi (Figure 4B). Intrigued by these data, we next tested whether perturbation of Rel itself might impact α-Syn levels. Indeed, RNAi-mediated knockdown of Rel resulted in reduced levels of α-Syn in the fly (Figure 4E), whereas its over-expression had the opposite effect (Figure 4F). Based on these outcomes, we conclude that AKT regulates the protein levels of α-Syn in a manner that is likely dependent on Rel. We also investigated whether α-Syn overexpression influences Rel levels. However, no changes in Rel protein levels were observed under conditions of α-Syn overexpression (Supplementary Figure 3).

We then explored whether modifying *Rel* impacts locomotor activity of α-Syn-expressing flies. *Rel* overexpression led to a higher proportion of female (Figure 4G, top panel) and male (Figure 4G, bottom panel) flies in the lower zones (zones 1 and 2), with fewer individuals in the upper zones (zones 4 and 5), indicating impaired locomotor activity. Conversely, RNAi-mediated knockdown of *Rel* significantly increased the proportion of flies in zone 5 and decreased the proportion in the lower zones (zones 1 and 2), in both sexes. These findings suggest that *Rel* not only influences α-Syn levels, but also plays a role in modulating locomotor activity, further supporting its involvement in the α-Syn-induced toxicity pathway.

**Figure 5:**
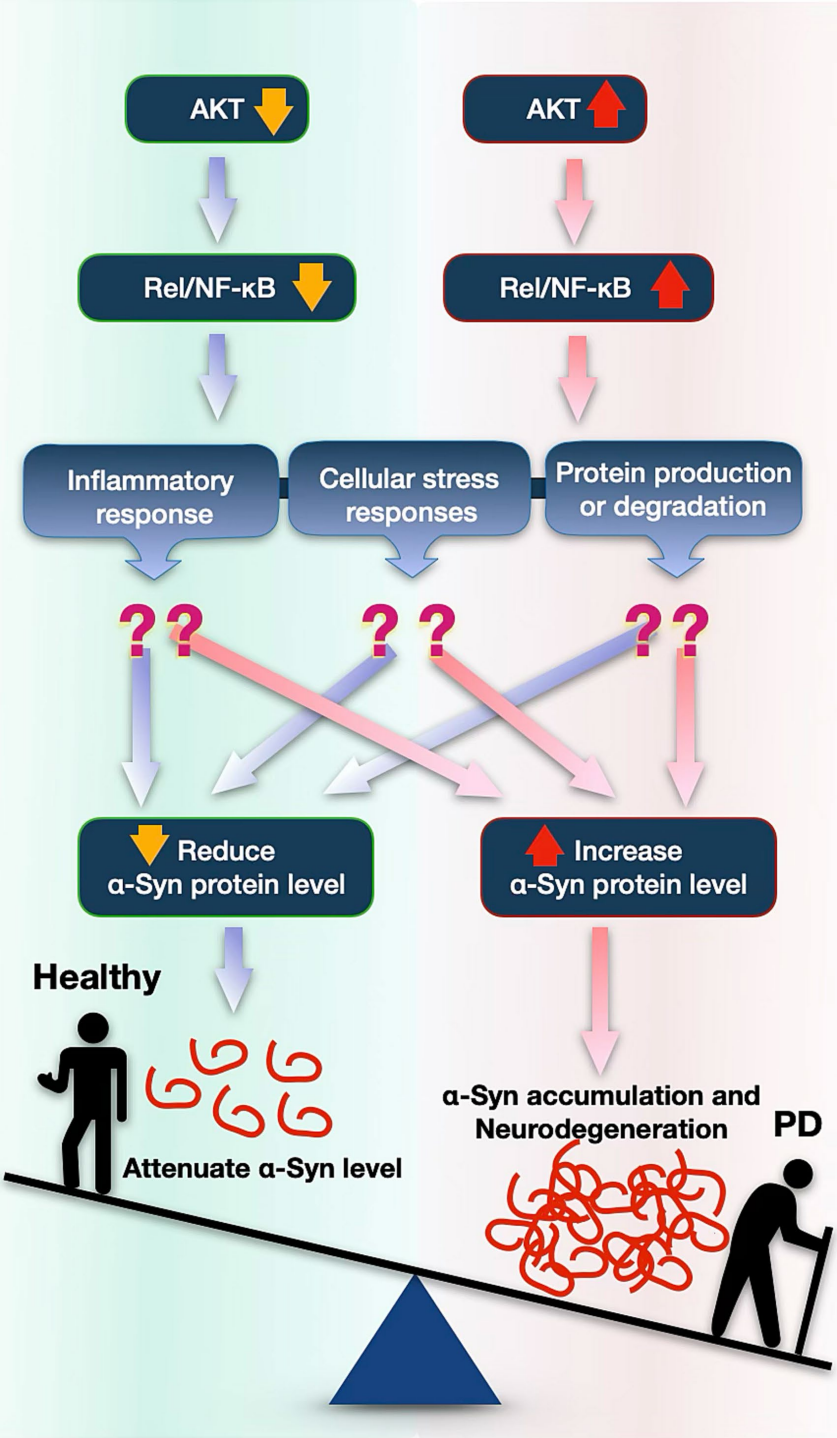
Proposed model illustrating how the AKT pathway might impact accumulation of α-Syn and its toxicity in PD. Reduced AKT expression decreases NF-κB (Rel in *Drosophila*) signaling, leading to lower α-Syn protein levels possibly by influencing protein degradation pathways, by inflammatory activation, or by other stress responses. Conversely, increased AKT and NF-κB is associated with elevated α-Syn accumulation, possibly contributing to neurodegenerative processes involved in PD.

Altogether, these data suggest that AKT affects α-Syn protein levels, perhaps through NF-κB (Rel). AKT and NF-κB emerge as potential targets for reducing α-Syn toxicity in PD (Figure 5).

## Discussion

Our study examined the ability of A-443654, an AKT inhibitor, to counteract α-Syn-induced toxicity in a *Drosophila* model of PD. Administering 1,000 μM A-443654 showed promising effects, extending lifespan and enhancing mobility in flies pan-neuronally expressing human α-Syn. This dosage is notably higher than the concentrations used in cell culture studies, where effective doses consisted of 0.3 μM in HEK-293 cells (Gandelman et al., 2021), 1.0 μM in MiaPaCa cells (Han et al., 2007), and 500 nM in hippocampal slices (Sun et al., 2016). Our initial trials using 1 μM, 10 μM, and 100 μM concentrations in α-Syn-expressing flies did not produce significant results (data not shown), leading us to test higher doses, which ultimately demonstrated efficacy. Unlike *in vitro* studies, where drug concentration and cellular uptake are tightly controlled, the fly model required administration through food, with 100 μL of the 1,000 μM solution applied on top of fly media. This method does not allow for precise tracking of drug intake, digestion, or absorption efficiency, which partially clarifies why we did not observe the same α-Syn reduction as reported in cell-based studies. This variation in drug delivery may also account for the milder effect of the compound on α-Syn toxicity *in vivo*. To address this limitation, we employed genetic approaches to knockdown AKT using RNAi (Figure 4), which effectively reduced AKT and α-Syn levels and demonstrated the therapeutic potential of AKT inhibition for alleviating α-Syn toxicity.

Our experiments revealed notable sex-specific differences (Figures 1, 2), with female flies consistently showing longer median survival and better locomotor performance compared to males. This observation aligns with previous studies in flies expressing various toxic proteins (Johnson et al., 2022; Sujkowski et al., 2024; Sujkowski et al., 2022; Patel et al., 2023). These differences may, in part, be attributed to physical factors such as the larger body size and potentially greater resilience of female flies. In human populations, PD is more prevalent among men, with approximately 65% of patients being male (Marras et al., 2018; Smilowska et al., 2024). Additionally, men are 1.5 times more likely to develop PD compared to women (Hirsch et al., 2016). These disparities underscore the importance of studying sex-specific differences, which may be influenced by genetic, hormonal, or transcriptional factors (Patel and Kompoliti, 2023). Although the precise mechanisms remain unclear, our findings highlight the need for further investigation into the cellular and molecular bases of sexual dimorphism in neurodegenerative diseases.

In addition to sexual dimorphism, we observed that treatment with A-443654 did not alter overall AKT protein levels (Figure 2D). This observation aligns with its mechanism of action. A-443654 functions as an ATP-competitive and reversible inhibitor targeting all three AKT isoforms (AKT1, AKT2, and AKT3) by binding to the ATP-binding site, thereby reducing AKT activity without altering its total protein levels. A-443654 treatment similarly showed no changes in total AKT protein levels in cellular models (Shi et al., 2005; Luo et al., 2005). Unfortunately, we were unable to quantify the percentage of AKT inhibition in our feeding experiments in flies due to the lack of antibodies capable of specifically detecting phosphorylated AKT or monitoring phosphorylation changes in AKT’s downstream targets in *Drosophila*. However, RNAi-mediated knockdown of AKT resulted in reduced levels of the downstream protein Rel, indirectly indicating suppression of the AKT pathway.

AKT overexpression resulted in a significant increase in α-Syn levels, suggesting a direct link between AKT activity and α-Syn accumulation. This notion underlines AKT signaling as a key regulator of α-Syn homeostasis *in vivo*, consistent with evidence that the PI3K/AKT pathway modulates protein degradation processes, including autophagy and the ubiquitin-proteasome system (Zhao et al., 2015; Morel et al., 2017; Xu et al., 2015), both critical for clearing misfolded and aggregated proteins (Webb and Brunet, 2014). AKT activation may suppress autophagy through the mTOR pathway (Manning and Toker, 2017; Zhao et al., 2015), reducing the degradation of α-Syn and promoting its accumulation; AKT inhibition could enhance autophagic clearance (Palmieri et al., 2017a,b; Pantazi et al., 2022), helping to lower α-Syn levels. Previous studies also suggested that the AKT inhibitor A-443654 reduces the transcription of doublecortin-like kinase 1 (Gandelman et al., 2021), an inhibitor of α-Syn lysosomal degradation (Vazquez-Velez et al., 2020). Together, these findings highlight the intricate relationship between AKT activity and α-Syn homeostasis. Further investigation is needed to fully understand the therapeutic potential of targeting AKT in managing α-synucleinopathies, where modulation of AKT may help prevent toxic protein accumulation and alleviate neurodegeneration in PD.

The AKT pathway plays a key role in regulating NF-κB activity (Bai et al., 2009). NF-κB is a transcription factor that regulates a wide range of cellular responses, including inflammation, immune response, and cell survival (Guo et al., 2024; Liu et al., 2017). When we knocked down AKT, we observed decreased protein levels of NF-κB. This led us to investigate whether lowering NF-κB levels could also influence α-Syn protein levels. Indeed, we found that NF-κB knockdown reduced α-Syn protein levels, whereas NF-κB overexpression led to an increase in α-Syn levels, suggesting that NF-κB is involved in α-Syn regulation.

AKT and NF-κB cascades are key players in cell survival signaling pathways, particularly in regulating autophagy and apoptosis (Tamatani et al., 1999; Chen et al., 2000). In PD and other neurodegenerative diseases, NF-κB is typically activated by cellular stressors (Wang et al., 2002), including the presence of misfolded proteins (Nivon et al., 2012) like α-Syn. Once activated, NF-κB translocates to the nucleus and upregulates pro-inflammatory cytokines, which can create a neuroinflammatory environment (Kaltschmidt et al., 2022). Recent studies indicate that α-Syn oligomers stimulate NF-κB activity in microglia and astrocytes, enhancing neuroinflammation and potentially accelerating neuronal damage (Leandrou et al., 2024). Inflammatory cytokines, such as IL-1β and TNF-α, promote α-Syn aggregation and Lewy body formation in neurons, establishing a feedback loop where α-Syn aggregates induce inflammation, which, in turn, promotes further aggregation (Sarkar et al., 2020; Codolo et al., 2013). Studies also found that α-Syn fibrils interact with immune receptors like TLR2 and TLR4 on microglia (Kim et al., 2021), triggering NF-κB activation (Li et al., 2021) and leading to increased cytokine production, which perpetuates this loop of aggregation and inflammation (Joers et al., 2024). Inhibition of NF-κB could potentially break this cycle, reducing α-Syn aggregation and improving cellular homeostasis. Thus, targeting both AKT and NF-κB may provide synergistic benefits, decreasing α-Syn accumulation and promoting α-Syn homeostasis. Our findings highlight the therapeutic potential of combined AKT and NF-κB inhibition in managing α-synucleinopathies, although further research is needed to refine these strategies and assess their safety and efficacy in neurodegenerative disease models.

A-443654 failed to confer protection in flies exposed to rotenone, a mitochondrial toxin that induces PD-like symptoms (Johnson and Bobrovskaya, 2015). This suggests that AKT inhibition may specifically counteract α-Syn-related pathologies rather than general PD mechanisms. Rotenone toxicity induces oxidative stress and mitochondrial dysfunction in dopaminergic neurons (Xiong et al., 2012; Johnson and Bobrovskaya, 2015) leading to increased NF-κB activity, which contributes to inflammatory responses and neuronal damage. NF-κB activation caused by rotenone can occur through both AKT-dependent (Farid et al., 2024) and AKT-independent (Gao et al., 2013) pathways, with the generation of reactive oxygen species (Morgan and Liu, 2011) and TLR (Toll-like receptor) signaling (Heidari et al., 2022) playing more prominent roles. As a result, AKT inhibition may exert limited protective effects in rotenone-treated models. Considering that dopaminergic neurons are primarily affected in PD, our findings should be further pursued in the future with a special focus on these types of neuronal cells. While our study utilized the elav-Gal4 driver to achieve pan-neuronal expression, subsequent research employing dopaminergic-specific drivers, such as Th-Gal4 or Ddc-Gal4 (Reiszadeh Jahromi et al., 2021), would provide valuable insights into the mechanisms underlying AKT’s role in α-Syn toxicity in DA neurons.

We additionally found that A-443654 was mildly protective in fly models of SCA2 (ataxin-2 Q117) and SCA3 (ataxin-3 Q80), where higher doses of A-443654 slightly alleviated toxicity from expanded polyglutamine repeats and extend the lifespan of the flies. These findings strengthen our hypothesis that A-443654 exerts its effects by modulating protein aggregation. In addition, studies in mouse models of genetic neurodevelopmental disorders showed that A-443654 can improve hippocampal synaptic plasticity (Sun et al., 2016), suggesting its potential as a therapeutic agent against memory impairments (Zhang et al., 2019). This raises the possibility that A-443654 counteracts neurodegeneration by enhancing synaptic plasticity across different types of neuronal cells. This hypothesis requires further investigation to fully understand its therapeutic potential in neurodegenerative contexts.

In summary, our findings revealed that AKT pathway modulation affects α-Syn levels and toxicity in a synucleinopathy-specific manner. These results underscore the therapeutic potential of targeting the AKT pathway in PD and related disorders. Future studies should further investigate the molecular mechanisms by which AKT influences α-Syn accumulation, particularly the roles of autophagy and ubiquitin-proteasome pathway in α-Syn clearance. Targeting this molecular mechanism could provide an alternative way to fight PD.

## Data Availability

The original contributions presented in the study are included in the article/Supplementary material, further inquiries can be directed to the corresponding authors.

## References

[ref1] Akinade T. C. Babatunde O. O. Adedara A. O. Adeyemi O. E. Otenaike T. A. Ashaolu O. P.. (2022). Protective capacity of carotenoid trans-astaxanthin in rotenone-induced toxicity in *Drosophila melanogaster*. Sci. Rep. 12:4594. doi: 10.1038/s41598-022-08409-4, PMID: 35301354 PMC8931097

[ref2] Bai D. Ueno L. Vogt P. K. (2009). Akt-mediated regulation of NfkappaB and the essentialness of NfkappaB for the oncogenicity of Pi3K and Akt. Int. J. Cancer 125, 2863–2870. doi: 10.1002/ijc.24748, PMID: 19609947 PMC2767458

[ref3] Benskey M. J. Perez R. G. Manfredsson F. P. (2016). The contribution of alpha synuclein to neuronal survival and function - implications for Parkinson's disease. J. Neurochem. 137, 331–359. doi: 10.1111/jnc.13570, PMID: 26852372 PMC5021132

[ref4] Blount J. R. Patel N. C. Libohova K. Harris A. L. Tsou W. L. Sujkowski A.. (2023). Lysine 117 on ataxin-3 modulates toxicity in Drosophila models of spinocerebellar Ataxia type 3. J. Neurol. Sci. 454:120828. doi: 10.1101/2023.05.30.542896 37865002 PMC10841544

[ref5] Brand A. H. Perrimon N. (1993). Targeted gene expression as a means of altering cell fates and generating dominant phenotypes. Development 118, 401–415. doi: 10.1242/dev.118.2.401, PMID: 8223268

[ref6] Bridi J. C. Hirth F. (2018). Mechanisms of alpha-Synuclein induced Synaptopathy in Parkinson's disease. Front. Neurosci. 12:80. doi: 10.3389/fnins.2018.00080, PMID: 29515354 PMC5825910

[ref7] Brunet A. Datta S. R. Greenberg M. E. (2001). Transcription-dependent and -independent control of neuronal survival by the Pi3K-Akt signaling pathway. Curr. Opin. Neurobiol. 11, 297–305. doi: 10.1016/S0959-4388(00)00211-7, PMID: 11399427

[ref8] Calabresi P. Mechelli A. Natale G. Volpicelli-Daley L. Di Lazzaro G. Ghiglieri V. (2023). Alpha-synuclein in Parkinson's disease and other synucleinopathies: from overt neurodegeneration back to early synaptic dysfunction. Cell Death Dis. 14:176. doi: 10.1038/s41419-023-05672-9 36859484 PMC9977911

[ref9] Chen C. Edelstein L. C. Gelinas C. (2000). The Rel/Nf-kappaB family directly activates expression of the apoptosis inhibitor Bcl-x(L). Mol. Cell. Biol. 20, 2687–2695. doi: 10.1128/MCB.20.8.2687-2695.2000, PMID: 10733571 PMC85484

[ref10] Codolo G. Plotegher N. Pozzobon T. Brucale M. Tessari I. Bubacco L.. (2013). Triggering of inflammasome by aggregated alpha-synuclein, an inflammatory response in synucleinopathies. PLoS One 8:e55375. doi: 10.1371/journal.pone.0055375, PMID: 23383169 PMC3561263

[ref11] Coulom H. Birman S. (2004). Chronic exposure to rotenone models sporadic Parkinson's disease in *Drosophila melanogaster*. J. Neurosci. 24, 10993–10998. doi: 10.1523/JNEUROSCI.2993-04.2004, PMID: 15574749 PMC6730201

[ref12] Cuervo A. M. Stefanis L. Fredenburg R. Lansbury P. T. Sulzer D. (2004). Impaired degradation of mutant alpha-synuclein by chaperone-mediated autophagy. Science 305, 1292–1295. doi: 10.1126/science.1101738, PMID: 15333840

[ref13] Dauer W. Przedborski S. (2003). Parkinson's disease: mechanisms and models. Neuron 39, 889–909. doi: 10.1016/S0896-6273(03)00568-3, PMID: 12971891

[ref14] Dong-Chen X. Yong C. Yang X. Chen-Yu S. Li-Hua P. (2023). Signaling pathways in Parkinson's disease: molecular mechanisms and therapeutic interventions. Signal Transduct. Target. Ther. 8:73. doi: 10.1038/s41392-023-01353-3, PMID: 36810524 PMC9944326

[ref15] Farid H. A. Sayed R. H. El-Shamarka M. E. Abdel-Salam O. M. E. El Sayed N. S. (2024). Pi3K/Akt signaling activation by roflumilast ameliorates rotenone-induced Parkinson's disease in rats. Inflammopharmacology 32, 1421–1437. doi: 10.1007/s10787-023-01305-x, PMID: 37541971 PMC11006765

[ref16] Feany M. B. Bender W. W. (2000). A Drosophila model of Parkinson's disease. Nature 404, 394–398. doi: 10.1038/35006074, PMID: 10746727

[ref17] Gallia G. L. Tyler B. M. Hann C. L. Siu I. M. Giranda V. L. Vescovi A. L.. (2009). Inhibition of Akt inhibits growth of glioblastoma and glioblastoma stem-like cells. Mol. Cancer Ther. 8, 386–393. doi: 10.1158/1535-7163.MCT-08-0680, PMID: 19208828 PMC4498795

[ref18] Gandelman M. Dansithong W. Kales S. C. Paul S. Maag G. Aoyama E.. (2021). The Akt modulator A-443654 reduces alpha-synuclein expression and normalizes Er stress and autophagy. J. Biol. Chem. 297:101191. doi: 10.1016/j.jbc.2021.101191, PMID: 34520759 PMC8482485

[ref19] Gao F. Chen D. Hu Q. Wang G. (2013). Rotenone directly induces Bv2 cell activation via the p38 Mapk pathway. PLoS One 8:e72046. doi: 10.1371/journal.pone.0085476, PMID: 23977201 PMC3748029

[ref20] Garcia-Reitbock P. Anichtchik O. Bellucci A. Iovino M. Ballini C. Fineberg E.. (2010). Snare protein redistribution and synaptic failure in a transgenic mouse model of Parkinson's disease. Brain 133, 2032–2044. doi: 10.1093/brain/awq132, PMID: 20534649 PMC2892942

[ref21] Gargano J. W. Martin I. Bhandari P. Grotewiel M. S. (2005). Rapid iterative negative geotaxis (Ring): a new method for assessing age-related locomotor decline in Drosophila. Exp. Gerontol. 40, 386–395. doi: 10.1016/j.exger.2005.02.005, PMID: 15919590

[ref22] Girish C. Muralidhara. (2012). Propensity of Selaginella delicatula aqueous extract to offset rotenone-induced oxidative dysfunctions and neurotoxicity in *Drosophila melanogaster*: implications for Parkinson's disease. Neurotoxicology 33, 444–456. doi: 10.1016/j.neuro.2012.04.002, PMID: 22521218

[ref23] Glaviano A. Foo A. S. C. Lam H. Y. Yap K. C. H. Jacot W. Jones R. H.. (2023). Pi3K/Akt/mtor signaling transduction pathway and targeted therapies in cancer. Mol. Cancer 22:138. doi: 10.1186/s12943-023-01827-6, PMID: 37596643 PMC10436543

[ref24] Goedert M. Jakes R. Spillantini M. G. (2017). The synucleinopathies: twenty years on. J. Parkinsons Dis. 7, S51–S69. doi: 10.3233/JPD-179005 28282814 PMC5345650

[ref25] Gomez-Benito M. Granado N. Garcia-Sanz P. Michel A. Dumoulin M. Moratalla R. (2020). Modeling Parkinson's disease with the alpha-Synuclein protein. Front. Pharmacol. 11:356. doi: 10.3389/fphar.2020.00356, PMID: 32390826 PMC7191035

[ref26] Guo Q. Jin Y. Chen X. Ye X. Shen X. Lin M.. (2024). Nf-kappaB in biology and targeted therapy: new insights and translational implications. Signal Transduct. Target. Ther. 9:53. doi: 10.1038/s41392-024-01757-9, PMID: 38433280 PMC10910037

[ref27] Han E. K. Leverson J. D. Mcgonigal T. Shah O. J. Woods K. W. Hunter T.. (2007). Akt inhibitor A-443654 induces rapid Akt Ser-473 phosphorylation independent of mtorc1 inhibition. Oncogene 26, 5655–5661. doi: 10.1038/sj.onc.1210343, PMID: 17334390

[ref28] Heidari A. Yazdanpanah N. Rezaei N. (2022). The role of toll-like receptors and neuroinflammation in Parkinson's disease. J. Neuroinflammation 19:135. doi: 10.1186/s12974-022-02496-w, PMID: 35668422 PMC9172200

[ref29] Hers I. Vincent E. E. Tavare J. M. (2011). Akt signalling in health and disease. Cell. Signal. 23, 1515–1527. doi: 10.1016/j.cellsig.2011.05.004, PMID: 21620960

[ref30] Hirsch L. Jette N. Frolkis A. Steeves T. Pringsheim T. (2016). The incidence of Parkinson's disease: a systematic review and meta-analysis. Neuroepidemiology 46, 292–300. doi: 10.1159/000445751, PMID: 27105081

[ref31] Hu D. Sun X. Liao X. Zhang X. Zarabi S. Schimmer A.. (2019). Alpha-synuclein suppresses mitochondrial protease ClpP to trigger mitochondrial oxidative damage and neurotoxicity. Acta Neuropathol. 137, 939–960. doi: 10.1007/s00401-019-01993-2, PMID: 30877431 PMC6531426

[ref32] Joers V. Murray B. C. Mclaughlin C. Oliver D. Staley H. E. Coronado J.. (2024). Modulation of cannabinoid receptor 2 alters neuroinflammation and reduces formation of alpha-synuclein aggregates in a rat model of nigral synucleinopathy. J. Neuroinflammation 21:240. doi: 10.1186/s12974-024-03221-5, PMID: 39334169 PMC11438102

[ref33] Johnson M. E. Bobrovskaya L. (2015). An update on the rotenone models of Parkinson's disease: their ability to reproduce the features of clinical disease and model gene-environment interactions. Neurotoxicology 46, 101–116. doi: 10.1016/j.neuro.2014.12.002, PMID: 25514659

[ref34] Johnson J. L. Huang W. Roman G. Costa-Mattioli M. (2015). Torc2: a novel target for treating age-associated memory impairment. Sci. Rep. 5:15193. doi: 10.1038/srep15193, PMID: 26489398 PMC4614817

[ref35] Johnson S. L. Prifti M. V. Sujkowski A. Libohova K. Blount J. R. Hong L.. (2022). Drosophila as a model of unconventional translation in spinocerebellar Ataxia type 3. Cells 11:1223. doi: 10.3390/cells11071223, PMID: 35406787 PMC8997593

[ref36] Johnson S. L. Ranxhi B. Libohova K. Tsou W. L. Todi S. V. (2020). Ubiquitin-interacting motifs of ataxin-3 regulate its polyglutamine toxicity through Hsc70-4-dependent aggregation. eLife 9:e60742. doi: 10.7554/eLife.60742, PMID: 32955441 PMC7505662

[ref37] Kaltschmidt B. Helweg L. P. Greiner J. F. W. Kaltschmidt C. (2022). Nf-kappaB in neurodegenerative diseases: recent evidence from human genetics. Front. Mol. Neurosci. 15:954541. doi: 10.3389/fnmol.2022.954541, PMID: 35983068 PMC9380593

[ref38] Kim C. Kwon S. Iba M. Spencer B. Rockenstein E. Mante M.. (2021). Effects of innate immune receptor stimulation on extracellular alpha-synuclein uptake and degradation by brain resident cells. Exp. Mol. Med. 53, 281–290. doi: 10.1038/s12276-021-00562-6, PMID: 33594256 PMC8080790

[ref39] Leandrou E. Chalatsa I. Anagnostou D. Machalia C. Semitekolou M. Filippa V.. (2024). Alpha-Synuclein oligomers potentiate neuroinflammatory Nf-kappaB activity and induce ca(v)3.2 calcium signaling in astrocytes. Transl. Neurodegener. 13:11. doi: 10.1186/s40035-024-00401-4, PMID: 38378800 PMC10880263

[ref40] Li Y. Xia Y. Yin S. Wan F. Hu J. Kou L.. (2021). Targeting microglial alpha-Synuclein/Tlrs/Nf-kappaB/Nlrp3 Inflammasome Axis in Parkinson's disease. Front. Immunol. 12:719807. doi: 10.3389/fimmu.2021.818487, PMID: 34691027 PMC8531525

[ref41] Liu T. Zhang L. Joo D. Sun S. C. (2017). Nf-kappaB signaling in inflammation. Signal Transduct. Target. Ther. 2:17023-. doi: 10.1038/sigtrans.2017.23, PMID: 29158945 PMC5661633

[ref42] Louis E. D. Mayer S. A. Noble J. M. (2022). Merritt's neurology. Philadelphia: Wolters Kluwer.

[ref43] Luo Y. Shoemaker A. R. Liu X. Woods K. W. Thomas S. A. De Jong R.. (2005). Potent and selective inhibitors of Akt kinases slow the progress of tumors in vivo. Mol. Cancer Ther. 4, 977–986. doi: 10.1158/1535-7163.MCT-05-0005, PMID: 15956255

[ref44] Manning B. D. Toker A. (2017). Akt/Pkb signaling: navigating the network. Cell 169, 381–405. doi: 10.1016/j.cell.2017.04.001, PMID: 28431241 PMC5546324

[ref45] Marras C. Beck J. C. Bower J. H. Roberts E. Ritz B. Ross G. W.. (2018). Prevalence of Parkinson's disease across North America. Npj. Parkinsons Dis. 4:21. doi: 10.1038/s41531-018-0058-0 30003140 PMC6039505

[ref46] Mohite G. M. Dwivedi S. Das S. Kumar R. Paluri S. Mehra S.. (2018). Parkinson's disease associated alpha-Synuclein familial mutants promote dopaminergic neuronal death in *Drosophila melanogaster*. ACS Chem. Neurosci. 9, 2628–2638. doi: 10.1021/acschemneuro.8b00107, PMID: 29906099

[ref47] Morel J. Palao J. C. Castells J. Desgeorges M. Busso T. Molliex S.. (2017). Regulation of Akt-mtor, ubiquitin-proteasome and autophagy-lysosome pathways in locomotor and respiratory muscles during experimental sepsis in mice. Sci. Rep. 7:10866. doi: 10.1038/s41598-017-11440-5, PMID: 28883493 PMC5589872

[ref48] Morgan M. J. Liu Z. G. (2011). Crosstalk of reactive oxygen species and Nf-kappaB signaling. Cell Res. 21, 103–115. doi: 10.1038/cr.2010.178, PMID: 21187859 PMC3193400

[ref49] Nitulescu G. M. Van De Venter M. Nitulescu G. Ungurianu A. Juzenas P. Peng Q.. (2018). The Akt pathway in oncology therapy and beyond (review). Int. J. Oncol. 53, 2319–2331. doi: 10.3892/ijo.2018.4597, PMID: 30334567 PMC6203150

[ref50] Nivon M. Abou-Samra M. Richet E. Guyot B. Arrigo A. P. Kretz-Remy C. (2012). Nf-kappaB regulates protein quality control after heat stress through modulation of the Bag3-HspB8 complex. J. Cell Sci. 125, 1141–1151. doi: 10.1242/jcs.091041, PMID: 22302993

[ref51] Palmieri M. Pal R. Nelvagal H. R. Lotfi P. Stinnett G. R. Seymour M. L.. (2017a). Corrigendum: mtorc1-independent Tfeb activation via Akt inhibition promotes cellular clearance in neurodegenerative storage diseases. Nat. Commun. 8:15793. doi: 10.1038/ncomms15793, PMID: 28607479 PMC5474731

[ref52] Palmieri M. Pal R. Nelvagal H. R. Lotfi P. Stinnett G. R. Seymour M. L.. (2017b). mtorc1-independent Tfeb activation via Akt inhibition promotes cellular clearance in neurodegenerative storage diseases. Nat. Commun. 8:14338. doi: 10.1038/ncomms14338, PMID: 28165011 PMC5303831

[ref53] Pantazi I. Papafragkos I. Kolliniati O. Lapi I. Tsatsanis C. Vergadi E. (2022). Akt inhibition promotes autophagy and clearance of group B Streptococcus from the alveolar epithelium. Pathogens 11:1134. doi: 10.3390/pathogens11101134, PMID: 36297190 PMC9611837

[ref54] Patel N. Alam N. Libohova K. Dulay R. Todi S. V. Sujkowski A. (2023). Phenotypic defects from the expression of wild-type and pathogenic Tata-binding proteins in new Drosophila models of spinocerebellar Ataxia type 17. G3 (Bethesda) 13:jkad180. doi: 10.1093/g3journal/jkad180 37551423 PMC10542169

[ref55] Patel R. Kompoliti K. (2023). Sex and gender differences in Parkinson's disease. Neurol. Clin. 41, 371–379. doi: 10.1016/j.ncl.2022.12.001, PMID: 37030964

[ref56] Paulson H. L. Perez M. K. Trottier Y. Trojanowski J. Q. Subramony S. H. Das S. S.. (1997). Intranuclear inclusions of expanded polyglutamine protein in spinocerebellar ataxia type 3. Neuron 19, 333–344. doi: 10.1016/S0896-6273(00)80943-5, PMID: 9292723

[ref57] Peng Y. Wang Y. Zhou C. Mei W. Zeng C. (2022). Pi3K/Akt/mtor pathway and its role in Cancer therapeutics: are we making headway? Front. Oncol. 12:819128. doi: 10.3389/fonc.2022.1070761, PMID: 35402264 PMC8987494

[ref58] Prifti M. V. Libohova K. Harris A. L. Tsou W. L. Todi S. V. (2022). Ubiquitin-binding site 1 of pathogenic ataxin-3 regulates its toxicity in Drosophila models of spinocerebellar Ataxia type 3. Front. Neurosci. 16:1112688. doi: 10.3389/fnins.2022.1112688, PMID: 36733922 PMC9887036

[ref59] Pulst S. M. Nechiporuk A. Nechiporuk T. Gispert S. Chen X. N. Lopes-Cendes I.. (1996). Moderate expansion of a normally biallelic trinucleotide repeat in spinocerebellar ataxia type 2. Nat. Genet. 14, 269–276. doi: 10.1038/ng1196-269, PMID: 8896555

[ref60] Reiszadeh Jahromi S. Ramesh S. R. Finkelstein D. I. Haddadi M. (2021). Alpha-Synuclein E46K mutation and involvement of oxidative stress in a Drosophila model of Parkinson's disease. Parkinsons Dis 2021:6621507. doi: 10.1155/2021/6621507, PMID: 34285796 PMC8275411

[ref61] Richardson K. Sengupta M. Sujkowski A. Libohova K. Harris A. C. Wessells R.. (2024). A phenotypically robust model of spinal and bulbar muscular atrophy in Drosophila. J. Neurosci. Res. 102:e25278. doi: 10.1002/jnr.25278, PMID: 38284836 PMC11237963

[ref62] Rocha S. M. Bantle C. M. Aboellail T. Chatterjee D. Smeyne R. J. Tjalkens R. B. (2022). Rotenone induces regionally distinct alpha-synuclein protein aggregation and activation of glia prior to loss of dopaminergic neurons in C57Bl/6 mice. Neurobiol. Dis. 167:105685. doi: 10.1016/j.nbd.2022.105685, PMID: 35257879 PMC9615439

[ref63] Rocha E. M. De Miranda B. Sanders L. H. (2018). Alpha-synuclein: pathology, mitochondrial dysfunction and neuroinflammation in Parkinson's disease. Neurobiol. Dis. 109, 249–257. doi: 10.1016/j.nbd.2017.04.004, PMID: 28400134

[ref64] Saravanan K. S. Sindhu K. M. Mohanakumar K. P. (2005). Acute intranigral infusion of rotenone in rats causes progressive biochemical lesions in the striatum similar to Parkinson's disease. Brain Res. 1049, 147–155. doi: 10.1016/j.brainres.2005.04.051, PMID: 15936733

[ref65] Sarkar S. Dammer E. B. Malovic E. Olsen A. L. Raza S. A. Gao T.. (2020). Molecular signatures of Neuroinflammation induced by alphaSynuclein aggregates in microglial cells. Front. Immunol. 11:33. doi: 10.3389/fimmu.2020.00033, PMID: 32082315 PMC7006296

[ref66] Shi Y. Liu X. Han E. K. Guan R. Shoemaker A. R. Oleksijew A.. (2005). Optimal classes of chemotherapeutic agents sensitized by specific small-molecule inhibitors of akt in vitro and in vivo. Neoplasia 7, 992–1000. doi: 10.1593/neo.05355, PMID: 16331885 PMC1502019

[ref67] Smilowska K. Pietrzykowski T. Owczarek A. J. Dorsey E. R. Bloem B. R. Van Wamelen D. J. (2024). The prevalence of Parkinson's disease in Poland: regional and sex-related differences. J. Parkinsons Dis. 14, 521–532. doi: 10.3233/JPD-230291, PMID: 38457147 PMC11091586

[ref68] Sokratian A. Zhou Y. Tatli M. Burbidge K. J. Xu E. Viverette E.. (2024). Mouse alpha-synuclein fibrils are structurally and functionally distinct from human fibrils associated with Lewy body diseases. Sci. Adv. 10:eadq3539. doi: 10.1126/sciadv.adq3539, PMID: 39485845 PMC11800946

[ref69] Spillantini M. G. Schmidt M. L. Lee V. M. Trojanowski J. Q. Jakes R. Goedert M. (1997). Alpha-synuclein in Lewy bodies. Nature 388, 839–840. doi: 10.1038/42166, PMID: 9278044

[ref70] Sujkowski A. Ranxhi B. Bangash Z. R. Chbihi Z. M. Prifti M. V. Qadri Z.. (2024). Progressive degeneration in a new Drosophila model of spinocerebellar ataxia type 7. Sci. Rep. 14:14332. doi: 10.21203/rs.3.rs-3592641/v1 38906973 PMC11192756

[ref71] Sujkowski A. Richardson K. Prifti M. V. Wessells R. J. Todi S. V. (2022). Endurance exercise ameliorates phenotypes in Drosophila models of spinocerebellar ataxias. eLife 11:e75389. doi: 10.7554/eLife.75389, PMID: 35170431 PMC8871352

[ref72] Sun J. Liu Y. Tran J. O’Neal P. Baudry M. Bi X. (2016). mtorc1-S6K1 inhibition or mtorc2 activation improves hippocampal synaptic plasticity and learning in Angelman syndrome mice. Cell. Mol. Life Sci. 73, 4303–4314. doi: 10.1007/s00018-016-2269-z, PMID: 27173058 PMC5056144

[ref73] Takahashi M. Kanuka H. Fujiwara H. Koyama A. Hasegawa M. Miura M.. (2003). Phosphorylation of alpha-synuclein characteristic of synucleinopathy lesions is recapitulated in alpha-synuclein transgenic Drosophila. Neurosci. Lett. 336, 155–158. doi: 10.1016/S0304-3940(02)01258-2, PMID: 12505616

[ref74] Tamatani M. Che Y. H. Matsuzaki H. Ogawa S. Okado H. Miyake S.. (1999). Tumor necrosis factor induces Bcl-2 and Bcl-x expression through NfkappaB activation in primary hippocampal neurons. J. Biol. Chem. 274, 8531–8538. doi: 10.1074/jbc.274.13.8531, PMID: 10085086

[ref75] Tanik S. A. Schultheiss C. E. Volpicelli-Daley L. A. Brunden K. R. Lee V. M. (2013). Lewy body-like alpha-synuclein aggregates resist degradation and impair macroautophagy. J. Biol. Chem. 288, 15194–15210. doi: 10.1074/jbc.M113.457408, PMID: 23532841 PMC3663539

[ref76] Vazquez-Velez G. E. Gonzales K. A. Revelli J. P. Adamski C. J. Alavi Naini F. Bajic A.. (2020). Doublecortin-like kinase 1 regulates alpha-Synuclein levels and toxicity. J. Neurosci. 40, 459–477. doi: 10.1523/JNEUROSCI.1076-19.2019, PMID: 31748376 PMC6948939

[ref77] Wang T. Zhang X. Li J. J. (2002). The role of Nf-kappaB in the regulation of cell stress responses. Int. Immunopharmacol. 2, 1509–1520. doi: 10.1016/S1567-5769(02)00058-9, PMID: 12433052

[ref78] Webb A. E. Brunet A. (2014). Foxo transcription factors: key regulators of cellular quality control. Trends Biochem. Sci. 39, 159–169. doi: 10.1016/j.tibs.2014.02.003, PMID: 24630600 PMC4021867

[ref79] Xiong N. Long X. Xiong J. Jia M. Chen C. Huang J.. (2012). Mitochondrial complex I inhibitor rotenone-induced toxicity and its potential mechanisms in Parkinson's disease models. Crit. Rev. Toxicol. 42, 613–632. doi: 10.3109/10408444.2012.680431, PMID: 22574684

[ref80] Xu D. Shan B. Lee B. H. Zhu K. Zhang T. Sun H.. (2015). Phosphorylation and activation of ubiquitin-specific protease-14 by Akt regulates the ubiquitin-proteasome system. eLife 4:e10510. doi: 10.7554/eLife.10510, PMID: 26523394 PMC4733041

[ref81] Zhang Y. Y. Liu M. Y. Liu Z. Zhao J. K. Zhao Y. G. He L.. (2019). Gpr30-mediated estrogenic regulation of actin polymerization and spatial memory involves Src-1 and Pi3K-mtorc2 in the hippocampus of female mice. CNS Neurosci. Ther. 25, 714–733. doi: 10.1111/cns.13108, PMID: 30714337 PMC6515707

[ref82] Zhao J. Zhai B. Gygi S. P. Goldberg A. L. (2015). Mtor inhibition activates overall protein degradation by the ubiquitin proteasome system as well as by autophagy. Proc. Natl. Acad. Sci. USA 112, 15790–15797. doi: 10.1073/pnas.1521919112, PMID: 26669439 PMC4703015

