# Supplementary material for: The effect of AKT inhibition in α-synuclein-dependent neurodegeneration
Source: Front Mol Neurosci. 2025 Feb 5;18:1524044. doi: 10.3389/fnmol.2025.1524044 (PMC11835820; doi:10.3389/fnmol.2025.1524044)

**Supplementary material:**

**The effect of AKT inhibition in α-synuclein-dependent neurodegeneration**

Bedri Ranxhi^1^, Zoya R. Bangash^1^, Zachary M. Chbihi^1^, Sokol V. Todi^1,2^, Peter A. LeWitt*^1,2,3^, Wei-Ling Tsou*^1^

1- Department of Pharmacology, Wayne State University School of Medicine

2- Department of Neurology, Wayne State University School of Medicine

3- Department of Neurology, Henry Ford Health Systems, Detroit, Michigan

*Correspondence:

wtsou@wayne.edu (Wei-Ling Tsou); aa1142@wayne.edu (Peter A. LeWitt)

540 E Canfield, Scott Hall Rm 3108, Detroit, MI 48201, USA.

Keywords: α-Synuclein, synucleinopathy, AKT, NF-κB, neurodegenerative disease, Parkinson’s disease, proteinopathy, protein misfolding disorders


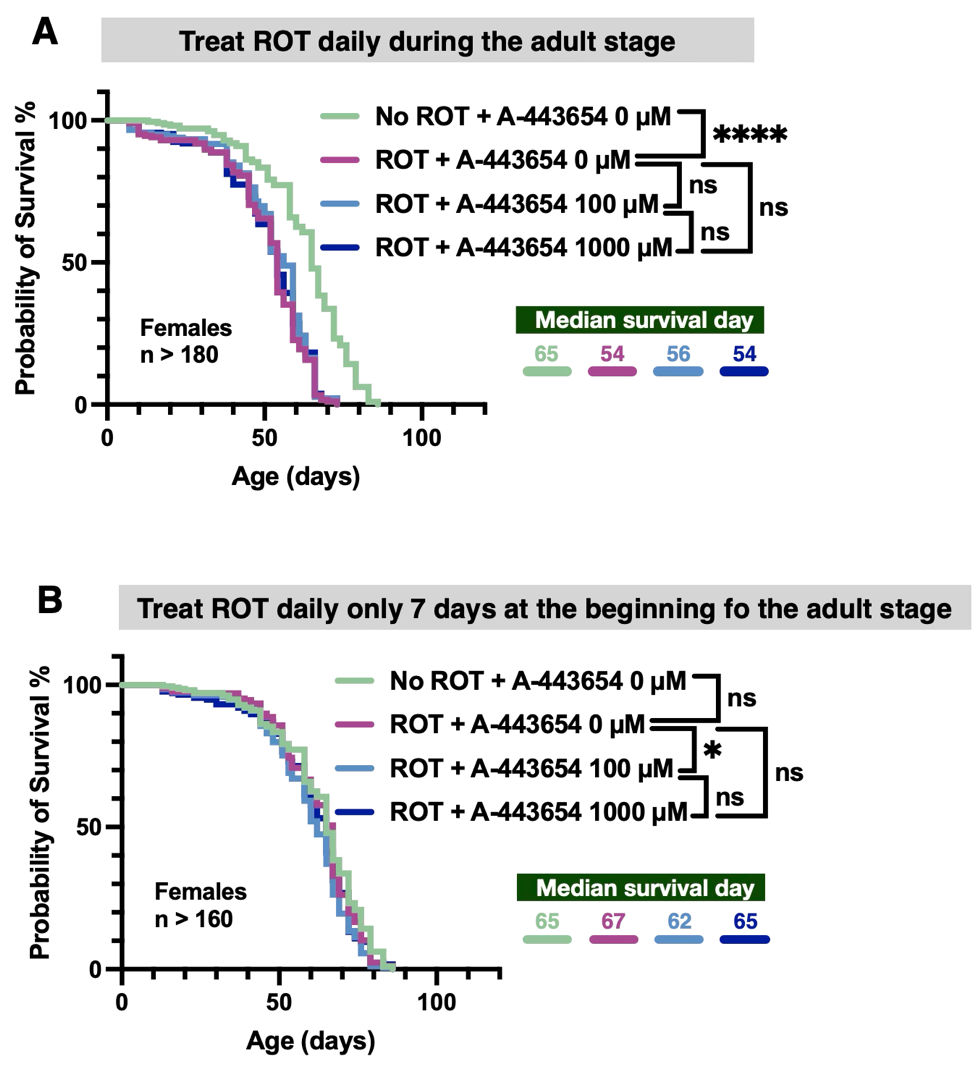


**Supplementary figure 1. The impact of A-443654 on ROT treated female flies**

A) Longevity of female flies in the rotenone (ROT)-feeding experiment, in which ROT was given continuously throughout the lives of flies, with A-443654 treatment commencing on day 7. B). Longevity of female flies in ROT-feeding experiment, in which ROT administration was halted on day 7 and A-443654 was introduced on day 7 for the remainder of the flies’ lifespan. Statistical analysis was conducted using log-rank tests; significance levels are denoted as follows: (ns) for no significant difference, (*) for p < 0.05, and (****) for p < 0.0001. N > 160 per group..


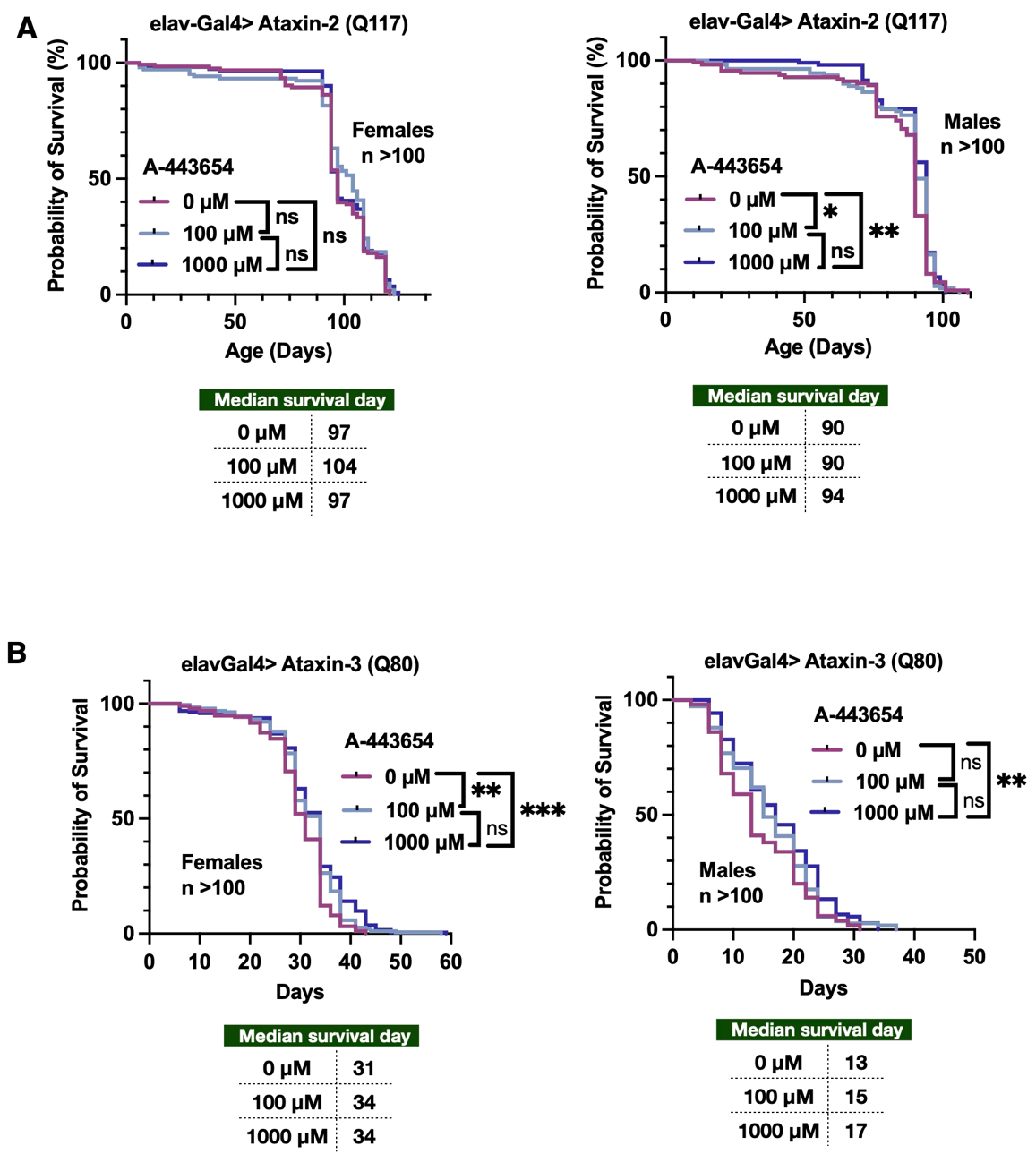


**Supplementary figure 2. Effect of A-443654 on the longevity of flies pan-neuronal expressing Ataxin-2-Q117 and Ataxin-3-Q80**

Longevity curves of adult female and male flies pan-neuronally expressing (A) Ataxin-2-Q117 and (B) Ataxin-3-Q80 during development and adulthood. Flies were fed with the indicated concentrations of A-443654. Statistical analysis was performed using log-rank tests. Significance levels are denoted as follows: (ns) for no significant difference, (*) for p<0.05, (**) for p<0.01, (***) for p<0.001and (****) for p<0.0001. Each group included N > 100 flies.


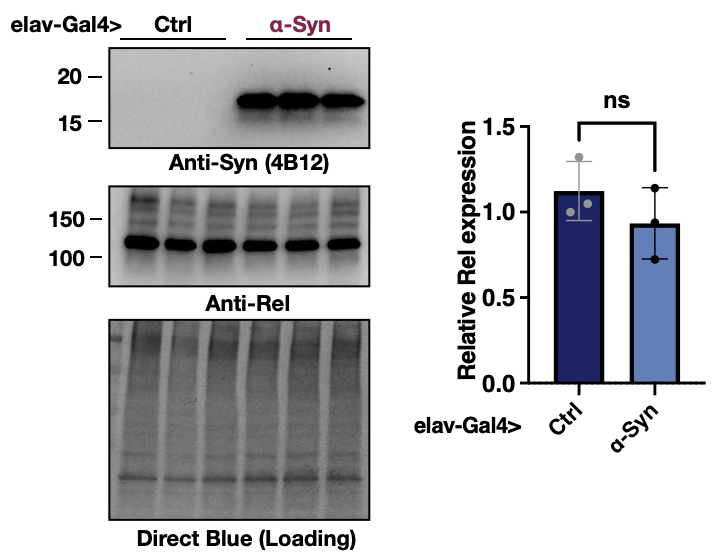


**Supplementary figure 3: The impact of α-Syn on Relish protein levels**

Western blot analysis of α-Syn and Relish protein levels in flies pan-neuronally expressing either a control or α-Syn. Statistical analysis was performed using Mann-Whitney one-tailed test, with "ns" indicating no significant difference. Data represent the mean ± SD, with N = 3 biological replicates per group.

**Supplementary figure 4. Uncropped western blot images**

**Uncropped figure 2D**


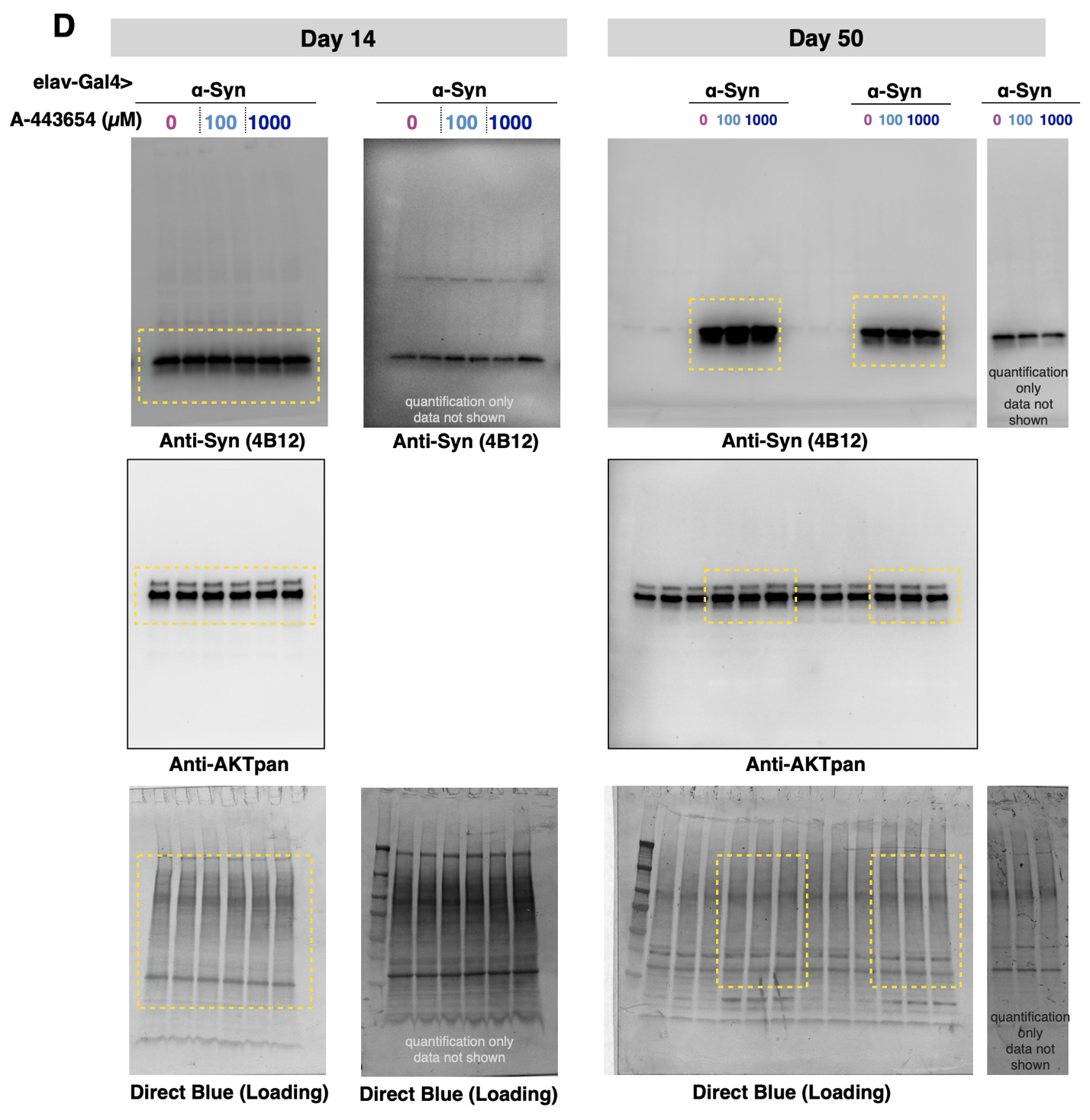


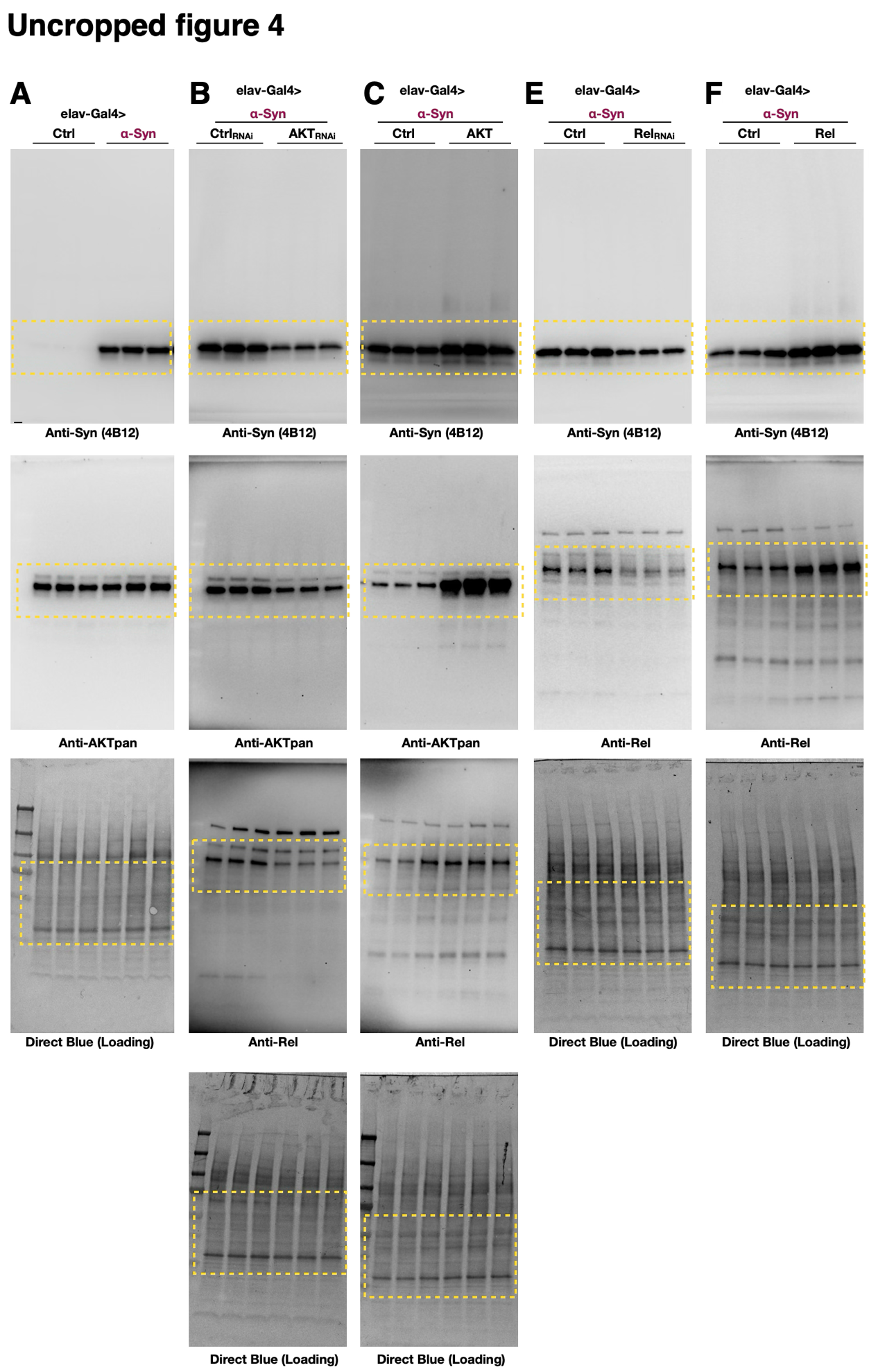


**Uncropped supplementary figure 3**


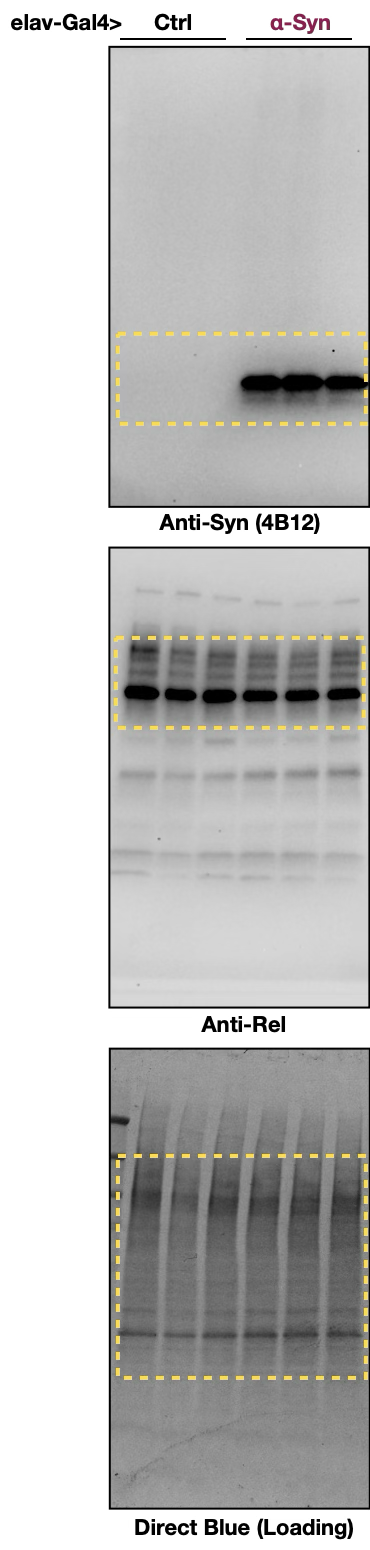

Supplement: Supplementary file 1 [file Table_1.DOCX]
